# Chinese Baijiu and Whisky: Research Reservoirs for Flavor and Functional Food

**DOI:** 10.3390/foods12152841

**Published:** 2023-07-26

**Authors:** Jinchen Li, Qiuyu Zhang, Baoguo Sun

**Affiliations:** 1 Key Laboratory of Geriatric Nutrition and Health, Beijing Technology and Business University, Ministry of Education, Beijing 100048, China; zqy21213@163.com (Q.Z.); sunbg@btbu.edu.cn (B.S.); 2 Key Laboratory of Brewing Molecular Engineering of China Light Industry, Beijing Technology and Business University, Beijing 100048, China; 3 Beijing Laboratory for Food Quality and Safety, Beijing Technology and Business University, Beijing 100048, China

**Keywords:** Chinese baijiu, whisky, food flavor, sensory analysis, functional food

## Abstract

Baijiu is a traditional spirit with high reputation in the Chinese community, and whisky, on the other hand, is a renowned spirit in Western culture, with both contributing a major proportion to the consumption and revenue in the global spirit market. Interestingly, starting with similar raw materials, such as grains, diverse production methods lead to different organoleptic profiles. In addition, such enormous attention they attract renders them as a crucial part in food and the related industry. Therefore, great efforts are made in improving product quality and optimizing production processes, such as flavor enhancement, facility development, and deep utilization of byproducts. Given the huge impacts and great involvements of these spirits in the general food industry, research focusing on either spirit is of referential significance for other relevant fields. With the aim of facilitating such collaboration, this review discusses the current research status, in a comparative manner, of both spirits in respect to key production processes-oriented sensory and flavor analysis, deep utilization of byproducts, and spirit-derived functional food investigations. Finally, the internal correlations based on the abovementioned criteria are identified, with research prospects proposed.

## 1. Introduction

Spirits, or liquors, make up essential parts of the global alcoholic drink industry, or even the broader food industry. Specifically, spirits, such as whisky, brandy, rum, vodka, gin, tequila, and Chinese Baijiu, are of great popularity. Starting from different raw materials, such as grains (whisky, vodka, gin, Chinese Baijiu), fruits (brandy, rum, gin, tequila), vegetables (vodka, gin), and sugars (vodka, rum, gin), each spirit embraces unique production techniques leading to distinctive flavor profiles. Among them, whisky and baijiu share the most similarities in terms of raw materials (grains) and production processes (saccharification, fermentation, distillation, aging, and blending) and are both popular and symbolic alcoholic drinks in both Western and Eastern cultures.

Baijiu, also known as Chinese national liquor, is a distilled spirit originating from China. Embracing a history of 2000 years, baijiu enjoys a high reputation in the Chinese community. It ranks at the top on the global spirit-consuming list, with ~7.5 billion liters consumed in China in 2021 [1], and therefore, the baijiu industry, as a part of the food sector, plays a key role in the Chinese national economy. Made from diverse grains as raw materials, such as sorghum, corn, barley, and rice, and different production methods, baijiu is known for various aroma types, of which the symbolic ones are strong, light, sauce, rice, and mixed flavors [2]. Unlike most globally renowned alcoholic beverages, production of baijiu is featured with solid-state fermentation prior to distillation, with multi-microbes involved (semi-solid-state and liquid fermentation are also applied in some cases). Therefore, it attracts great research interest due to the complex liquor-making processes and the resultant intricate flavor profiles. Such studies have been reviewed thoroughly [2,3,4] to encourage further research on elucidating the correlations between baijiu flavors and the underlying microbial networks.

On the other side of the world, whisky, as a major global spirit, receives much attention, both commercially and academically. Reported with an annual consumption volume of ~6.0 billion liters globally in 2021 (Statista data), whisky has long been one of the most popular spirits worldwide. Similar to baijiu, whisky is known for different types, such as Scotch, Irish, American, Canadian, and Japanese whisky, resulting from different raw materials and/or production methods. On the other hand, however, uniqueness is noted especially for whisky flavors.

Both baijiu and whisky start with grains as raw materials and proceed with malting and saccharifying to produce fermentable sugar, followed by alcoholic fermentation. Distillation is conducted as the next step, yielding spirits, and final products are not released until aging for most types of baijiu and all whisky. However, major differences, especially flavors, are noticed between these two spirits. Critical processing steps, such as saccharification, fermentation, and aging, are essential in determining final flavors, and these are also where the variances occur for these two spirits. In addition, both products are made with such complex raw materials, as well as processing steps, that numerous compounds, contributing to the general properties of the spirits, are present. Among these, compounds of bio-functional values present in spirits are investigated as well, such as peptides, polysaccharides, and phenols. It may be controversial to study the health potentials of alcoholic beverages; however, such studies are necessary in that certain compounds in these spirits may mitigate, to various degrees, the side effects brought by ethanol. In addition, such investigation sheds light on wider ranges of studies focusing on functional food with biological values.

In this review, thorough discussions about baijiu and whisky, the two most popular and similar spirits representative of either Eastern or Western culture, are made in a comparative manner, focusing on flavors and health potentials. Similarities and differences are summarized, with underlying connections elucidated. New insights on future research derived from both spirits in terms of sensory-oriented food flavor analysis, deep utilization of food byproducts, and bio-functional food exploration are proposed, in the hope of inspiring more related research.

## 2. Sensory-Based Flavor Characteristics

The organoleptic property, as a common first impression of any food product, is among the most critical parameters during food evaluation. Though recent decades have witnessed great developments on instrumental analysis targeting flavor compounds, sensory evaluation is essential in identifying complex interactions among different compounds displaying unique flavor characteristics. The flavor wheel, a useful tool in interpreting flavor quality in an intuitive manner, has been well adapted for both the whisky and baijiu industries.

**Figure 1:**
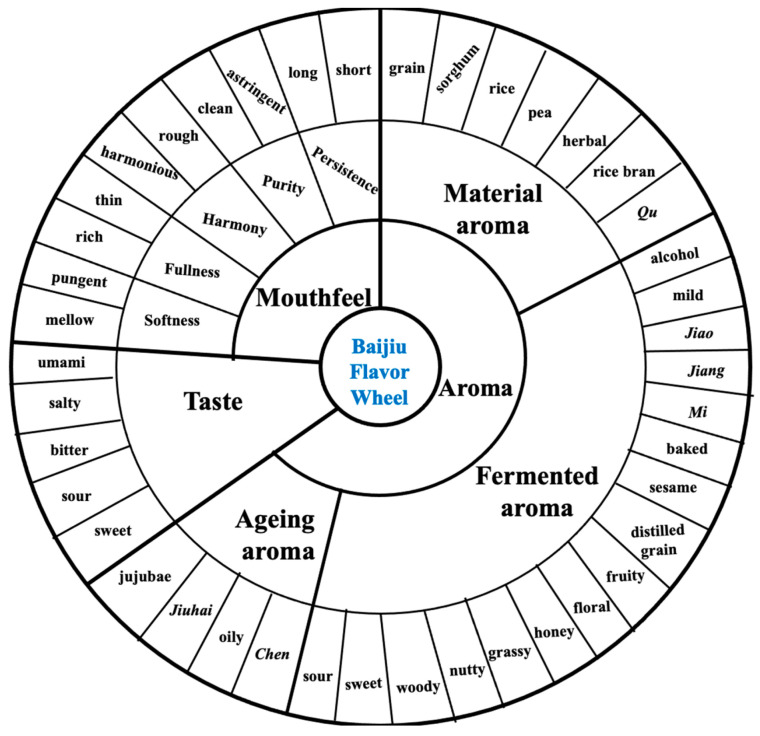
Baijiu flavor wheel, adapted from the Chinese National Standard of Terminology of Baijiu sensory evaluation. Explanations of certain phrases: *Qu* (fermentation and saccharification agent comprised of mixtures of micro-organisms; detailed explanation available in Section 3.1), *Jiao* (aromas derived from specific fermentation vessels, which are the habitat of mixtures of micro-organisms and grains, characteristic aromas in strong-aroma baijiu), *Jiang* (sauce aromas derived from unique production techniques characteristic of sauce-aroma baijiu), *Mi* (aromas derived from baijiu made from rice instead of sorghum), *Chen* (aromas derived from baijiu aging), *Jiuhai* (a specific aging vessel; detailed explanation available in Section 3.2).

For baijiu (Figure 1), the wheel consists of 3 first, 9 s, and 42 third tiers of sensory descriptors, elucidating the major aroma, mouthfeel and taste characters of baijiu. The majority of flavors are ingredient- and fermentation-derived, such as rice; herbal, as of material aromas; and sesame, fruity, and floral, as of fermentation aromas. The aging process for baijiu, however, is different from that of whisky and imparts little influence on flavors from storage vessels. On the other hand, different sensory characters are summarized for diverse aroma types, including sauce-, strong-, light-, and rice-aroma baijiu as the four major aroma types of baijiu. For sauce-aroma type baijiu, common aroma descriptors include sauce-like, roasted, caramel, nutty, floral, and fruity [3,5,6]. It is typically sweet with a prominent sauce flavor on the palate, followed by a delicate and long aftertaste [7]. Strong-aroma type, on the other hand, features with a strong, yet mellow and fragrant, aroma profile, full-bodied, soft, and sweet on the palate, and finishes with a long and pure aftertaste [7,8]. As for light-aroma type, it is commonly described with a pure and mild aroma profile, mellow and sweet on the palate, followed by a refreshing aftertaste [3,7]. Finally, rice-aroma types are generally recognized as rice-scented, featured with sweet and mellow, refreshing, and mouth-drooling tastes [7].

**Figure 2:**
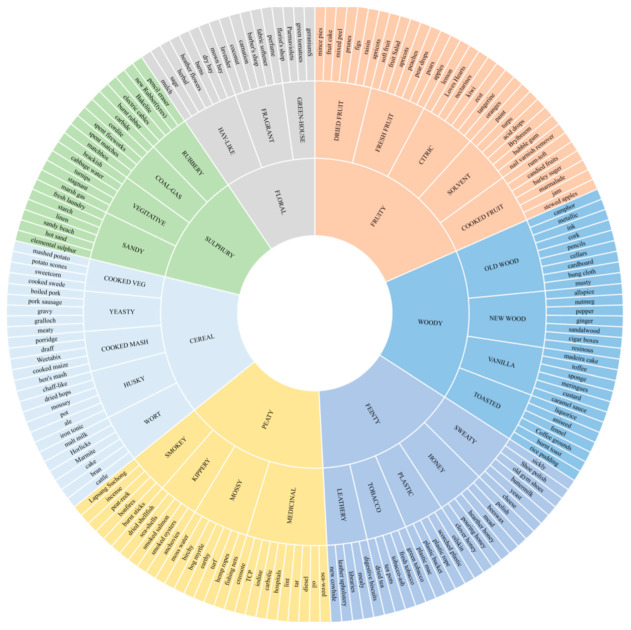
Whisky flavor wheel.

As for whisky, the major differences compared with baijiu lie within certain production steps, namely, fermentation, and aging. Various versions of flavor wheels have been generated according to the whisky types, as well as the purpose. For malt whisky, sensory descriptors are categorized into eight groups, including flavors derived from fermentation, distillation, and maturation (Figure 2). Grain-based aromas originally arise from raw materials and are further modified by fermentation and distillation, while fruity and floral aromas are mainly derived from fermentation and distillation. Peaty character is commonly imparted by malt kilning and is a symbolic scent of many malt whiskies. Feints and sulphury compounds come in during distillation, and the aromas evolve as the distillation progresses. Such characters may as well be mellowed and transformed during aging in good wooden vessels. In addition, maturation in various styles of barrels bring in woody and winey aromas, which are typical aging characters of whisky. Other whiskies, on the other hand, such as Bourbon, of which the dominant ingredient is maize, is endowed with flavor profiles with subtle differences. For instance, unlike maize, wheat, and malt, which bring in sweet notes to the spirits, rye typically imparts spicy character. For Bourbon whiskey, flavors categorized as spices, sweet, and earthy are generally present, acting as distinctive notes for Bourbon compared with malt whisky.

Moreover, alcohol content (alcohol strength) is also a crucial parameter of spirit products, not only as a physicochemical property, but also an interactive factor with flavor and sensory profiles. For whisky, 36–50% ABV (Alcohol by Volume) covers most products, where 40–43% ABV is most often seen in markets. On the other hand, wider alcohol strength ranges apply for baijiu, from 18 to 78% ABV, where 38–55% ABV is more common. Ethanol is an essential factor for aroma perception in the headspace above the spirit, and sensorial effects brought by different ethanol contents are worth studying. However, such reports are limited, therefore presenting a potential research gap for future research. Nevertheless, a recent study on the impact of whisky dilution on aroma perception indicated that the usual consumption behavior of adding water to whisky does make a difference. Specifically, dilution over 80% whisky/20% water may significantly reduce the differences among various styles, and yet, differences were observed between Scotch and American styles as further dilution continued. This study provided crucial insights into interactions of spirit dilution and aroma perception and was of referential value for further investigations for both spirits.

## 3. Key Processing Steps Associated with Flavors

**Figure 3:**
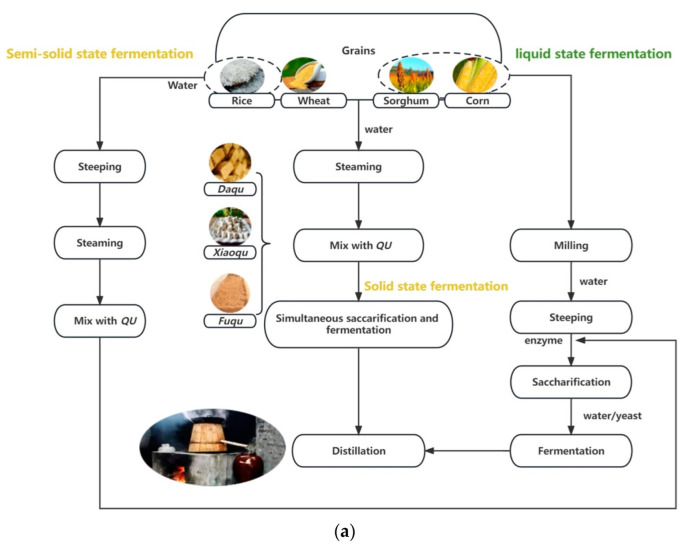

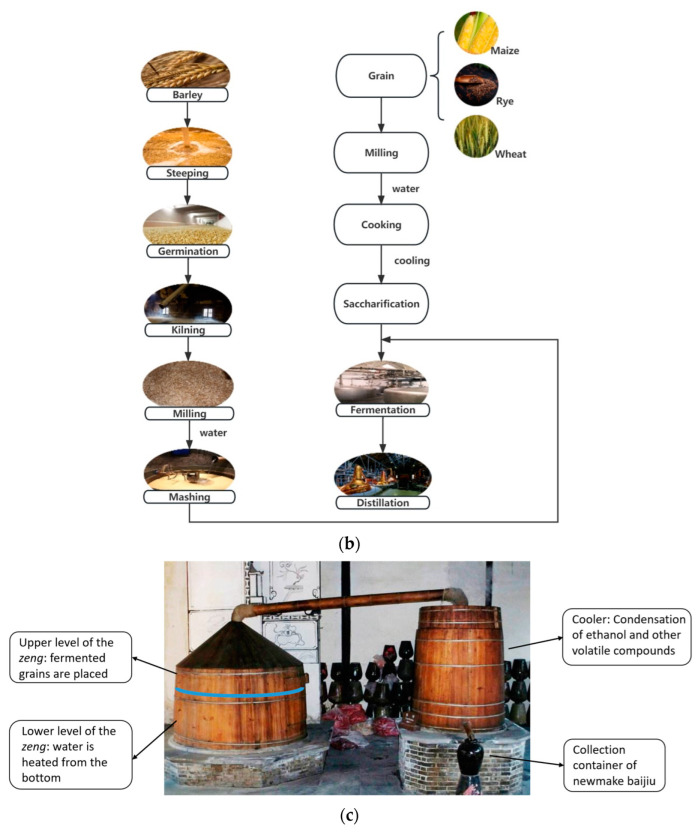
Simplified illustration flowchart of baijiu (**a**) and whisky (**b**) production procedures prior to aging. Simplified illustration of traditional *zeng* (**c**). Explanations of *Qu*, *daqu*, *xiaoqu*, and *fuqu* and *zeng* can be found in Section 3.1 and Section 3.2.

As has been mentioned, the production methods of both spirits are similar in general, including the processing of raw materials, fermentation, distillation, and aging. However, as traditional alcoholic beverages in different cultures with long histories, both baijiu and whisky possess complex production procedures (Figure 3), of which a few candidate processes make huge differences in the styles, flavors, and quality of the final products.

### 3.1. Microbial Diversity Involved in Saccharification and Fermentation

Grains, rich in starch as carbohydrates, are the starting point of making both spirits, and yet diverse flavor profiles are obtained from them. Saccharification and alcoholic fermentation are two of the major steps that make the difference. For whisky production, these two steps happen in a stepwise manner. In most cases, such as Scotch whisky, saccharification is initiated by endogenous enzymes in malts, including α-amylase and β-amylase. Potent enzymatic activities are induced in malted barley by steeping, kilning, milling, and mashing. During the mashing process, starch-degrading enzymes work together to produce maltose from starch. Specifically, α-amylase helps with the liquefication of the gelatinized starch molecules, while β-amylase proceeds further with hydrolysis, yielding maltose [9]. In addition, other enzymes, such as carboxypeptidases responsible for the production of amino acids, are also developed in the starchy endosperm. The abovementioned enzymes facilitate the production and extraction of fermentable sugars, amino acids, vitamins, and minerals from malts, so that the following alcoholic fermentation can readily occur. For whiskies made from other grains, including corn, rye, and wheat, of which amylolytic enzymes are limited in the raw materials, malted barleys are normally added to provide the “diastatic power”, thus producing fermentable sugars. For instance, a typical Bourbon mash contains ~70% corn, 15% rye, and 15% malted barley, of which starch-degrading enzymes are present, with amino acids and small assimilable peptides contributed by other cereals. Commercial enzyme preparations may be applied for certain types of whiskies, of which malt is absent from the mash bills. Such examples include Canadian rye whisky, where milled rye with maize and/or wheat are used as raw materials, and enzymes, like amylases with different temperature tolerance, β-glucanases, and amyloglucosidases, are added at different stages of mashing to ensure the optimal release of fermentation ingredients.

Fermentation occurs after mashing when the cereal/malt worts are cooled down, and yeasts (typically *Saccharomyces cerevisiae*) are inoculated. Yeasts consume sugars and nitrogenous compounds during alcoholic fermentation, yielding ethanol, CO_2_, and secondary metabolites, including volatile compounds, and such compounds play a crucial role in the flavor profiles of both new-make spirits and final products. As is known, yeast-derived aroma profiles are species/strain-dependent, and therefore, each distillery possess their unique yeast selections to produce whiskies of individual styles. The application of “non-conventional” yeasts, mainly non-*Saccharomyces* yeasts, which is a common practice in winemaking, is gradually attracting attention, and distinctive flavors are obtained for these trials [10,11]. In addition, since boiling is absent prior to fermentation, the non-sterile environments after wort production enable the presence of diverse local microflora, such as lactic acid bacteria (LAB). Among them, species of *Lactobacillus* are normally in dominance due to their tolerance of high alcohol content and acidic and anaerobic environments. Other surviving LABs include *Lactococcus*, *Pediococcus*, *Streptococcus*, and *Leuconostoc*. Typically recognized as spoilage in beer brewing, the limited occurrence of bacterial cultures and wild yeasts in whisky production is gradually deemed related to the improved flavor quality of the new-make spirit [12]. If present, they can produce lactic and acetic acid that can be esterified into ethyl lactate and ethyl acetate, imparting fruity, sweet, and creamy characteristics [13,14]. Additionally, γ-dodecalactone contributing the sweet and fatty flavors is also produced by LABs [14,15]. Therefore, lactic acid fermentation is sometimes desirable in distilleries. For instance, during whiskey production in the United States, spent mash (processed grains) can be added back, as a small portion, into the wort prior to fermentation, allowing the growth of lactic acid bacteria [16]. However, the occurrence and prosperity of the bacterial community during fermentation is crucial, since early growth may lead to poor ethanol yields. Alcoholic fermentation for whisky production generally lasts for 2–3 days, producing a final ethanol concentration of ~6–8% in the fermented worts/mashes, which are ready for the following distillation [9].

Unlike whisky, sorghum is one of the most popular raw materials in baijiu production, followed by corn, rice, wheat, millet, and peas. All these grains are rich in starch, and therefore, similar to whisky production, conversion of starch into fermentable sugars is an essential step. And, in fact, saccharification and fermentation are deemed the most critical for baijiu production, responsible for producing thousands of flavor compounds that make up the complex aroma profiles of baijiu. Solid-state fermentation, of which simultaneous saccharification and fermentation occur in a solid mixture, is a characteristic step in baijiu production responsible for the rich and complex flavors in final products. In addition, semi-solid-state and liquid-state saccharification and spontaneous fermentation are both typical, though less common, in baijiu production, and are reported as producing spirits with less aromatic profiles, yet with higher fermentation efficiency.

*Qu*, as the traditional saccharifying and fermentation agent, comprises tremendous amounts of microbes and enzymes, and can be divided into *daqu*, *xiaoqu*, *fuqu*, and others. *Daqu* is a brick-shaped solid mixture of grounded barley, wheat, peas, and various indigenous microbes, such as molds (*Mucor racemosus*, *Aspergillus niger*, *Thermomyces lanuginosus*), yeasts (*Saccharomyces cerevisiae*, *Candida*), bacteria (*Bacillus subtilis*, lactic acid bacteria, acetic acid bacteria), and actinomycetes [4]. Different cultivation temperatures result in corresponding *daqu* types, including high- (60–70 °C), medium- (50–60 °C), and low-temperature (~50 °C) *daqu*, leading to various flavor characteristics [3]. High-temperature *daqu* is typically applied to produce sauce-aroma baijiu and has been reported in relation with the production of phenylethanol, 1,3-butanediol, acetic acid, propanoic acid, methyl ester, guaiacol, and tetramethylpyrazine [4]. Medium-temperature *daqu* contributes a fuller flavor and taste and is often used in the production of strong-aroma baijiu, rich in phenylethanol, caproic acid, tetradecanoic acid ethyl ester, ethyl caproate, guaiacol, pyrazines, and caryophyllene [4]. Low-temperature *daqu*, on the other hand, comprises larger microbiota, leading to a higher fermentation efficiency than the two other types. Such an agent is typically used in the production of light-aroma baijiu and is responsible for a pure flavor and taste, of which phenylethanol, (Z)-2-octen-1-ol, hexanal, (E)-2-octenal, nonanoic acid, acetic acid, lactic acid, hexyl acetate, ethyl acetate, and pyrazines are characteristic flavor compounds resulting from low-temperature *daqu* [4]. Apart from *daqu*, *xiaoqu* and *fuqu* are two major saccharification and fermentation agents used in baijiu production, resulting in diverse flavor characters. *Xiaoqu* is composed of rice, herbs, and sometimes clay as the raw materials, and rich in indigenous microorganisms, mainly molds and yeasts, such as *Rhizopus* sp., *Rhizopus oryzae*, *Rhizopus peka*, and *S. cerevisiae* [4]. Baijiu produced by *xiaoqu* is known for light and fragrant aromas. Unlike those that harbor naturally occurring microbes, *fuqu*, starting from bran as the raw material, is made by deliberate inoculation of molds under optimal conditions, and therefore, is known for the best saccharification and fermentation capacity [3].

### 3.2. Distillation and Aging

Baijiu distillation is another characteristic feature in baijiu production, which is different from that of whisky. Solid-state fermentation is commonly seen in baijiu-making, and therefore, the key feature in distillation is to distill ethanol and flavor compounds out of solids, namely, fermented grains. Water vapor is, therefore, applied as the agent to bring out these compounds from the solids. *Zeng*, a huge pot where fermented grains are distilled, is typically used for the distillation vessel, especially for solid-state fermentation. Fermented grains are placed as the top layer in *zeng*, and water is heated from the bottom of the vessel, underneath fermented grains, resulting in the rise of water vapor to pass through the fermented solids and bring volatile compounds, such as ethanol and esters, out of it. These compounds are further condensed, producing spirits with plentiful compounds of flavor and other benefits. *Zeng* is more like a special steaming tower, in that fermented grains are placed layer by layer in *zeng*, and therefore, volatile compounds are vaporized and condensed repeatedly as they pass through each layer, achieving multi-distillation. The alcohol contents of new-make baijiu vary and is as high as 75% ABV.

Whisky distillation, on the other hand, however, is more well-known and has been extensively reviewed and discussed. Pot distillation and continuous distillation are major methods, and similar to baijiu, timings for heads, hearts, and feints selection are critical for both spirits’ production.

Aging, however, is also where the differences lie, and is critical for quality improvement for both spirits. New-make baijiu is described with odors like green, harsh, peppery, and raw cardboard [17]. For baijiu, aging typically happens in various vessels, including pottery jars, *jiuhai* (Mare Nectaris, a traditional aging container woven from vines and coated with hemp paper, animal blood, and lime), and stainless-steel vessels. During aging, various chemical reactions occur within the liquid, such as reduction, oxidation, esterification, hydrolysis, and the Maillard reaction, and compounds of low boiling points contributing negative flavor properties are eliminated by evaporation through micropores [18]. Oxidation and esterification reactions were confirmed by experimental investigations. During baijiu aging, the ester concentration increases due to the conversion of alcohols into their corresponding acids and esters, while a constant balance of ester levels is obtained via esterification [19]. Studies, with the help of density functional theory and molecular dynamics simulation, suggest that for strong-aroma type baijiu, the activation energy of acid hydroxyl-oxygen protonation was higher than that of ester alkyl-oxygen protonation, therefore explaining the increased level of acids and decreased ester concentration during aging in pottery jars [20]. In addition, the sorption effect is also proposed, especially when aged in pottery jars, and trials, like applying pottery chips, have been conducted, confirming the potential sorption effects [21]. Since pottery jars are reusable, such pottery materials are identified as vectors responsible for transferring flavor compounds into newly distilled baijiu and, therefore, accelerating the aging process [21].

Aging in wooden casks plays a crucial role in determining the flavors and styles of whisky. For many production regions, it is mandatory for new-make whisky spirit to be matured in wooden (mainly oak) containers for a period of time before being ready for markets. In Scotland, whiskies are required to be aged in oak barrels for at least 3 years, and it is not rare to find 12-year-old or even older products. The flavors and textures imparted to the spirits are time- and wood-dependent, leading to a grand selection of aging vessels for the worldwide distilleries. For Scotch whisky, it is a common practice to employ used barrels for maturation. For instance, the Sherry cask, which has previously been used for Sherry wine (a type of fortified wine) aging, is frequently applied by many whisky producers including Scotch whiskies for complex flavor extraction. And, such used barrels previously containing other alcoholic products, such as port (a type of fortified wine), madeira (a type of fortified wine), and Tokaji (a type of sweet wine made from grapes infected with noble rot), are popular choices for many producers. On the other hand, however, Bourbon whiskey only allows for the use of new oak barrels for maturation, while used Bourbon barrels can be reused by other production regions for more flavor blending and creation. Investigation aiming at characterizing different barrels revealed the chemical signatures of two popular options, Bourbon and Sherry casks [22]. Bourbon casks are identified by compounds, including flavonols, oligolignols, and fatty acids, whereas for Sherry casks, polyphenol glycosides, such as quercetin-glucuronide, myricetin-glucoside, and carbohydrates, can be used for discrimination [22].

Similar to some types of baijiu, whisky aging exposes the liquid to a micro-oxygen environment, owing to the permeation effect of the wood staves [23]. And, therefore, chemical reactions, such as oxidation, occur, enabling whisky stabilization and flavor formation. However, compounds transferred from wood into spirits and the following fusion are other major reasons shaping the characters. The frequently used oak wood normally comprises cellulose (45–50%), hemicellulose (22–25%), lignins (23–32%), and extractables (mainly acids, carbohydrates, and phenolic compounds, 3–10%) [24]. Oak woods of different origins are reported with dissimilar extractable properties. For instance, European oak is reported with higher amounts of phenolic compounds and extractable solids than American oak, whereas the latter is normally known for its greater contribution of oak flavor to liquids [24]. Sensory-directed study coupled with instrumental analysis identified ellagitannin vescalagin, castalagin, grandinin, roburins A-E, and 33-deoxy-33-carboxyvescalagin as the major compounds contributing to the astringency from the European oak (*Quercus alba* L.) [25]. The migration of extractable carbohydrates is elucidated in Bourbon whiskey [26]. Recalcitrant cellulose and hemicellulose are liberated from the barrel staves, and meanwhile, the spirit expands into and contracts from the wood, therefore forming a complex, which may be associated with the characteristic flavor and palate [26].

## 4. Volatile Compositions of Baijiu and Whisky

**Figure 4:**
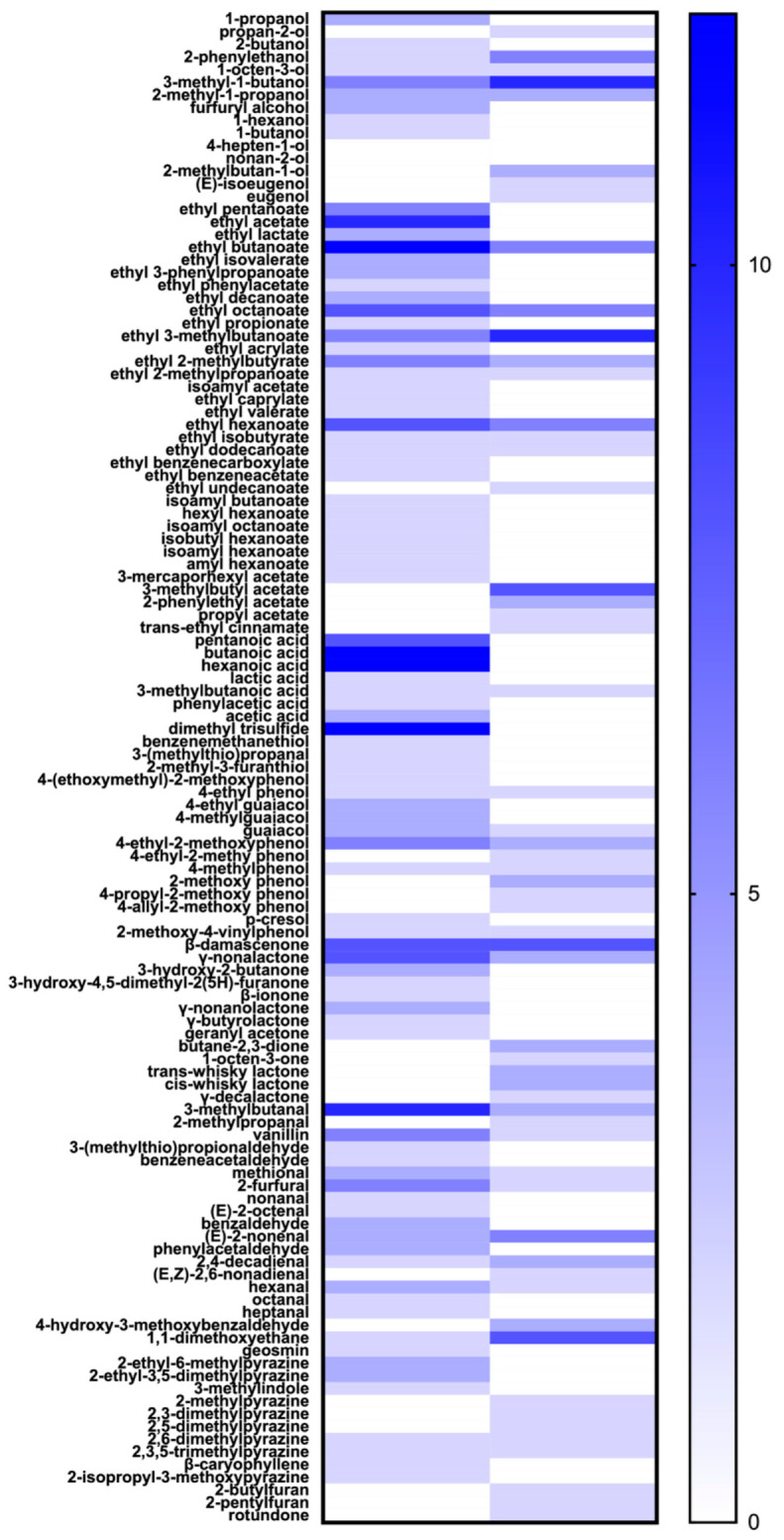
Heatmap of the occurrences of key aroma compounds identified in baijiu and whisky.

**Figure 5:**
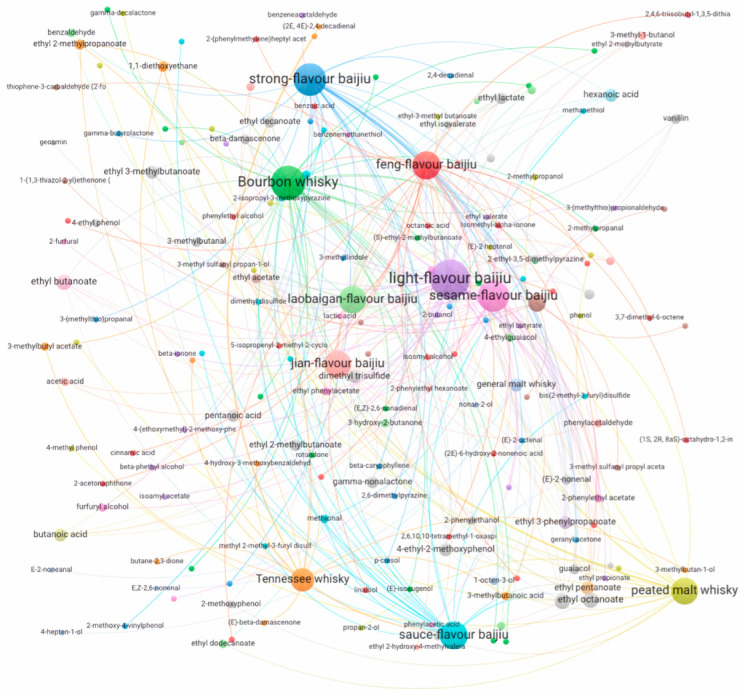
Correlations among different styles of baijiu and whisky based on volatile compositions.

With diverse sensory profiles identified for both spirits, it becomes a crucial task to reveal the scientific nature, namely, volatile compositions, to thoroughly understand the flavor and sensory characteristics. Such knowledge may, therefore, play an instructive role in quality improvement during production. Similarities are observed for both baijiu and whisky based on volatile components, while differences in terms of quantities and compositions contribute greatly to their distinctive sensory characters. For better data interpretation, a heatmap (Figure 4) was hereby generated in terms of the occurrences of key aroma compounds reported in baijiu and whisky, while a correlation network (Figure 5) based on volatile compositions was proposed for both spirits [5,8,18,25,27,28,29,30,31,32,33,34,35,36,37,38,39,40,41,42,43,44,45,46,47,48,49]. Data were obtained from various brands and styles of both spirits, with diverse sample extraction methods and identification methods applied in different studies. Such extraction methods include direct injection (DI), liquid–liquid extraction (LLE), liquid–liquid microextraction (LLME), solid-phase extraction (SPE), solid-phase microextraction (SPME), stir bar sorptive extraction (SBSE), and simultaneous distillation and extraction (SDE), and identification methods include GC-MS, GC-O-MS, GC-FID (flame ionization detector), GC × GC-TOFMS, GC-PFPD (pulsed flame photometric detection), GC-QTOF-MS, and UPLC-MS. For both spirits, factors such as alcohol strength, style, and production of origin, play crucial roles in volatile compositions and flavor profiles, making it complex to assess flavor similarities and differences. And, therefore, markers of significant representativeness are valuable for spirit comparison. Key aroma compounds, which are further identified in these studies by analyzing volatile concentrations and sensory contributions by means of the molecular sensory science approach, were included as such crucial markers for comparison due to their significant contributions to sensory properties (Figure 4). Specifically, the general methods involved in molecular sensory science study include identification and quantification of volatile compounds, aroma extract dilution analysis (AEDA) coupled with GC-O, aroma reconstitution, and omission tests coupled with sensory evaluation. And, such a comprehensive process renders it as a valid method for the identification of key aroma compounds, and therefore, comparisons accordingly help minimize the effects brought by other variables. Though both start with grains as raw materials, low similarities (~25%) in terms of key aroma compounds were observed in the baijiu and whisky ever reported, which may be explained by the diverse production methods of each spirit. On the other hand, a correlation network was proposed by analyzing volatile compositions available from the literature for both spirits (Figure 5). Surprisingly, certain styles of both spirits showed relevance, such as Bourbon whisky vs. most baijiu, and sauce-flavor baijiu vs. most whisky. To further specify the similarities and differences, detailed discussions are hereby made for common and crucial volatile compounds, including alcohols, esters, aldehydes, acids, and sulfur compounds.

### 4.1. Alcohols, Esters, Aldehydes

Esters are crucial volatile groups in almost all alcoholic beverages, endowing the liquids with diverse aroma characters, such as fruity, floral, sweet, and milky. Previous research illustrates the significant proportions of these compounds in both baijiu and whisky. For instance, a total of 510 types of esters were identified in baijiu [50], and such systematic investigations are also encouraged for whisky flavor research. The most common esters encountered in baijiu are ethyl esters, followed by methyl esters, propyl esters, butyl esters, amyl esters, hexyl esters, and lactones. Similarly, ethyl esters are the major ester groups as key aroma compounds present in whisky. Ethyl acetate and ethyl lactate are the most frequently observed esters in baijiu, and are present in all 12 aroma types of baijiu. Furthermore, all these aroma types share several other esters, including ethyl butyrate, ethyl valerate, ethyl caproate, ethyl heptanoate, ethyl caprylate, ethyl nonanoate, ethyl decanoate, propyl caproate, ethyl 2-methylpropionate, ethyl 2-methylbutyrate, ethyl 3-methylbutyrate, 3-methylbutyl caproate, ethyl phenylacetate, and diethyl succinate. Differently, ethyl 3-methylbutanoate is commonly identified as a major aroma-active compound in whisky, with whisky lactone being the characteristic ester in whisky.

Esters are produced via various reactions, including microbial metabolic synthesis and spontaneous chemical reactions. In addition, a few esters are ingredient-derived [50], which are applicable for both spirits owing to the similar ingredients used for production. Nevertheless, a group of esters, whisky lactones, is unique to whisky under the influence of aging in oak barrels. For both spirits, microbial enzymatic esterification accounts for the major source of ester compounds. Such a reaction is catalyzed by enzymes, including carboxylic acid hydrolase, lipase, esterase, and cutinase [51]. Due to the traditional manufacturing method, a complex microbial network is identified in baijiu production, thus harboring numerous microorganisms with versatile metabolic abilities, including esterification. A fungus, Monascus purpureus, recognized for high ester-producing ability, was isolated from baijiu fermentation starter. And, the key enzyme, a carboxylesterase, was identified to synthesize fatty acid esters [52]. Such an environment not only provides various microbes for complex aroma production, but acts as a microbial niche for screening microorganisms of potential benefit.

For whisky, it is noted that long-chain ethyl esters play a crucial role in shaping diverse aromas, though with similar compound compositions [53]. These compounds are produced from ethanol and medium-chain fatty acids during yeast fermentation. The number of the carbon numbers in the aliphatic chain of ethyl esters can be as many as 18 [54]; commonly occurring examples include ethyl hexanoate, ethyl octanoate, ethyl decanoate, ethyl dodecanoate, ethyl tetradecanoate, and ethyl hexadecanoate [53]. Research indicated that such hydrophobic esters, such as ethyl octanoate, ethyl decanoate, and ethyl dodecanoate, make up a great proportion of whisky headspace, are present at a significant quantity, and lead to key contributions to whisky aroma profiles [55,56].

### 4.2. Acids

Acids, in both volatile and non-volatile forms, are mainly produced during fermentation, the results of microbial metabolism. Specifically, they are produced by acetyl-transferase systems present in different microorganisms. For instance, byproducts including fusel alcohols are synthesized during ethanol production, and such alcohol compounds are further transformed to corresponding acids by alcohol dehydrogenases and aldehyde dehydrogenases. Examples include 2-methyl-1-propanoic acid and 3-methyl-1-butanoic acid. Additionally, for baijiu, Acetobacter aceti and lactic acid bacteria (LAB), responsible for the production of acetic acid and lactic acid, respectively, are commonly occurring microbes during fermentation. On the other hand, however, LAB may also be present in whisky fermentation in some cases. The late phase of lactic fermentation is mostly desirable in producing flavor-positive compounds, and yet, comprehensive mechanisms therein remain fully elucidated [57].

These compounds are extensively studied in many alcoholic drinks, including baijiu and whisky, from the perspective of flavor contribution. In addition, acids are also important compounds of health potential that are discussed in further details in the following sections. In baijiu, acids are frequently detected as metabolites of the complex microbial network involved in baijiu-making, whereas such compounds are rarely reported as key flavor compounds in whisky. Acids are present in baijiu at large ranges, from the highest of 65% for strong-aroma to as low as 9% for buckwheat-aroma. Major acids in baijiu include acetic acid, propionic acid, butanoic acid, hexanoic acid, pentanoic acid, heptanoic acid, nonanoic acid, octanoic acid, decanoic acid, 2-methylpropionic acid, and 3-methylbutanoic acid [58]. On the other hand, 3-methylbutanoic acid present in Tennessee whiskey is one of the few odor-active acids reported in whisky [28]. Despite the fact that acids are not as influential as other aroma compounds, such as esters, in determining volatile profiles of these spirits, yet their role as precursors of ester formation is non-neglectable. For instance, short-chain ethyl esters, which are responsible for fruity and floral characters, are formed via chemical reactions of short-chain acid compounds and ethanol. Furthermore, aroma-neutral acids can be transformed to flavor-active acids via chemical reactions during fermentation, including 2-methylpropanoic acid, 3-methylbutanoic acid, sorbic acid, lactic acid, and benzoic acid.

In addition, non-volatile acids play crucial roles in shaping the overall flavor characters by matrix effects, such as masking or synergistic effects. A study aiming at reconstituting the signature flavors of Laobaigan-aroma baijiu revealed the significance of these acids in altering the volatile profiles of the spirit matrices [44]. Matrices containing both non-volatile acids and odor-active compounds are judged as having elevated characters of fruity, acidic, floral, jujube, and grain, yet with reduced sweety and alcoholic notes, compared with those containing only odor-active compounds [44]. Specifically, lactic acid showed additive or synergistic odor effects on ethyl lactate and ethyl acetate by decreasing olfactory thresholds significantly [38]. Though acids are not among the top focus lists of whisky researchers, similar flavoromics-based interactive studies are encouraged to extensively depict the flavor map, as well as flavoring mechanism of whisky.

### 4.3. Sulfur Compounds

Sulfur compounds are a group of flavor substances crucial to both spirits. They can be a double-edged sword and offer pleasant aromas at low concentrations, yet make negative contributions at high levels. Certain sulfur compounds are identified as the key aroma compounds characteristic of some aroma-type baijiu. And, such examples include dimethyl sulfide and benzothiazole, responsible for smoky and toasty notes in strong-aroma baijiu [2]. Therefore, targeted research was conducted extensively for thorough understanding of the distribution of these sulfur-containing volatile compounds in final spirit products, as well as obtaining rational management through production for a more desirable flavor profile.

In whisky, sulfur compounds evolve throughout the whole production process. Dimethyl sulfide (DMS) is reported to be formed during the malting process. Specifically, DMS is produced during the kilning process from S-methyl methionine, which is originally formed from methionine during barley germination [59]. The higher the kilning temperature, the more DMS is produced. The following fermentation step leads to more production of sulfur compounds (Table 1), many of which derive from the methionine biosynthesis pathway. Similarly, for baijiu production, volatile sulfur compounds are mainly produced during fermentation, as well. And, yet, unlike the germination process for whisky, microbial hydrolysis of proteins naturally occurring in raw materials, such as sorghum, typically takes place for baijiu production, yielding sulfur-containing amino acids as precursors. Such compounds are further converted to various sulfur volatiles, including methional and dimethyl trisulfide [60]. In addition, the complex microbial system involved in baijiu production enables the production of other sulfur compounds, such as benzothiazole [61]. Moving forward, distillation is also a critical step in managing sulfur contents in the spirits, especially for whisky production. Copper is commonly acknowledged as the suitable material for the distillation apparatus, making it possible for undesirable sulfur removal, according to the longstanding industry practice. However, further investigations suggest the possibility of formation of sulfur volatiles by catalysis of copper compounds. Specifically, methyldisufanylmethane (dimethyl disulfide, DMDS) and methyl trisulfanylmethane (dimethyl trisulfide, DMTS) are proposed to be produced more in the presence of copper, with precursors being methanethiol, and methanethiol and hydrogen sulfide, respectively [62,63,64]. Finally, levels of certain sulfur compounds decrease during the maturation process for whisky. And, such compounds include DMDS, DMS, and DMTS [62]. The mechanisms therein remain unsolved, while the absorption effect brought by the inside charcoal layer of the oak may be one of the possibilities, which requires further scientific explanations.

Volatile sulfur compounds can be undesirable sometimes, especially for whisky. And, therefore, efforts are made for sulfur elimination. Physical treatment, with no chemical reactions involved, is recognized as a crucial solution as downstream management. Among them, charcoal or activated carbon are commonly used, though at the expense of potentially losing desirable components, due to the lack of selectivity in absorption. Different activated carbon materials were trialed, such as coal and coconut shell-based activated granular carbons, and reduced levels of sulfur volatiles were noted based on organoleptic characters. Further study indicated the possible relationship of pore size, contact duration, and contents of sulfur compounds is required [65].

## 5. Instrumental Analysis Methods

Volatile detection is one of the most crucial methods for thoroughly understanding the flavor profiles of both baijiu and whisky. However, challenges are present due to the following reasons: a. the complex matrix various products offer; b. the generally broad and vague reference parameters for certain volatiles based on the literature, such as the retention index, thus potentially leaving out novel compounds; c. the relatively high detection thresholds of many instruments for trace compounds.

Many challenges were addressed throughout the development of instrumental analysis, thus rendering it a useful tool for decoding the chemical composition of different spirits. For instance, specific detection methods for sulfur-, nitrogen-, and phosphorous-containing volatiles are utilized, including the nitrogen chemiluminescence detector (NCD), nitrogen phosphorous detector (NPD), sulfur chemiluminescence detector (SCD), and flame photometric detector (FPD). In addition, other applicable extraction and detection methods are devised and summarized in Table 2.

Despite analytical technologies advancing greatly, a fixed and mature methodology is yet absent for most types of baijiu and whisky. And, such conditions in turn encourage further technical improvement of volatile analysis to expand the identification scope, as well as the detection level of aroma compounds, especially those present at trace levels, yet potentially crucial to sensory profiles.

For baijiu, various extraction methods coupled with qualitative and quantitative approaches were trialed and revealed the occurrence of more than 1, 900 flavor compounds. And, these extraction methods include direct injection (DI), liquid–liquid extraction (LLE), liquid–liquid microextraction (LLME), solid-phase extraction (SPE), solid-phase microextraction (SPME), stir bar sorptive extraction (SBSE), and simultaneous distillation and extraction (SDE). Similarly, a few of the abovementioned extraction methods are applied in whisky flavor analysis, such as SPME and LLE. On the other hand, however, solvent-assisted flavor evaporation (SAFE) was also reported as one of the techniques used during aroma profiling. Such a method may help with the extraction of non-volatile compounds or compounds with lower boiling points migrated from oak barrels into whisky.

The following analytical processes are also customized based on the detection requirements of various volatile groups and spirit types. Common techniques include gas chromatographic mass spectrometry (GC-MS), ultra-high-performance liquid chromatography (UPLC), and two-dimensional gas chromatography time-of-flight mass spectrometry (GC×GC-TOF-MS). In analyzing particular compounds, such as compounds at trace levels, customized detection techniques are applied, such as nuclear magnetic resonance spectrometry (NMR), GC-NCD, GC-SCD, GC-NPD, GC-FPD, UPLC-high resolution mass spectrometry (UHPLC-HRMS), and UHPLC-quadrupole orbitrap HRMS (UHPLC-Q-Orbitrap HRMS).

Since volatile compositions are not in direct correlations with sensory characters due to the various thresholds and complex sensorial interactions between each compound, identifying the key aroma compounds contributing the crucial organoleptic properties is therefore of great importance. The molecular sensory approach, which incorporates instrumental analysis with sensory study, becomes increasingly popular for both baijiu and whisky research. Such a systematic methodology starts with the identification and quantification of volatile compounds, followed by recognizing their aroma contributions, via instrumental-coupled sensorial analysis. In identifying odor active values (OAVs), along with the conduction of aroma extract dilution analysis (AEDA), aroma omission, and reconstitution analysis, volatile compounds of crucial organoleptic contributions are identified for both spirits, which practically makes it possible for future potential flavor-targeted quality improvement.

## 6. Health Potentials

Both spirits share a common dilemma where large quantities of byproducts are obtained during the production process. Specifically, two byproducts are involved in the production of whisky, namely, draff and pot ale. Draff is the solid waste (wet grains) and is rich in carbohydrates, fiber, and proteins, whereas pot ale refers to the liquid waste from the distillation, rich in proteins and potentially copper, if copper still is used. Similarly, two major byproducts are identified from baijiu production, as well, *jiuzao* and *huangshui*, which are solid and liquid wastes, respectively. *Jiuzao* is the spent grains after distillation rich in proteins and carbohydrates. *Huangshui* is the viscous liquid produced from solid-state fermentation and, therefore, contains numerous trace compounds generated during complex fermentation. Currently, the solid wastes derived from both spirits’ production are mainly treated as animal feed and biomass combustion, while liquid wastes are discharged as waste water after minor treatment. However, in accordance with global carbon neutrality, such waste management is no longer suitable, and therefore, efforts are being made in exploring reusable solutions to bring out the full potential of these byproducts.

To begin with, the hidden values of these products are investigated thoroughly. Though utilizing similar raw materials as a start, various follow-up procedures for either spirit making, i.e., saccharification and fermentation, can potentially lead to diverse major components of health benefits. For baijiu, both *jiuzao* and *huangshui* are identified as valuable pools for potentially valued extractables, such as peptides and polysaccharides. Essential activities, such as antioxidant, anti-inflammatory, and immunomodulatory capacities, were identified within these compounds via in vitro assays (Table 3). Similarly, phenolic compounds, as a major group of compounds in grains, were studied in both whisky draff and baijiu *jiuzao*, where antioxidant capacity, to various degrees depending on specific compounds, was identified (Table 3). Rather than simply treated as feedstocks and fuels, such materials can be fully utilized for their biological potential, and further function-based investigation is encouraged. Subsequent exploitation may include production of functional foods or adding back to spirits, the latter of which requires additional research on their influence on the overall flavor profiles.

### 6.1. Within the Spirits

In addition to the byproducts, the health and functional values within the spirit are worth studying, as well. Such a topic is controversial since ethanol is deemed risky to the human body, and yet, both baijiu and whisky are mixed solutions containing thousands of trace compounds, therefore providing potential values, or mitigating the harmfulness ethanol brings. One of the major advantages that is generally acknowledged is the pleasure and psychological comforts it brings after proper intake. Further in-depth research targeting both physiological and psychological benefits are conducted for comprehensive explanation. Specifically, health potentials are discussed in respect to compounds by categories within the spirits and broad health values based on clinical surveys.

#### 6.1.1. Acids and Esters

Acids and esters are crucial flavor compounds present in both spirits, of which esters are widely identified as key odor compounds contributing to characteristic flavor profiles. Their occurrences in both spirits being elucidated, many of these compounds are investigated for their bioactive benefits.

Short-chain acids and their corresponding esters are proved to alleviate alcohol intoxication, and such effects are demonstrated on acetic acid and ethyl acetate [58]. Specifically, ethanol metabolism is altered and acute alcoholic liver injury is relieved by both compounds. In addition, reduction of intestinal inflammation is identified with acetate, propionate, and butyrate, with butyric acid being elucidated as a cancer therapeutic agent [58]. Short-chain aliphatic esters play crucial roles in the potentiation of the response of the GABAA (type A of γ-aminobutyric acid) receptor, and such activity is helpful in releasing emotional relaxation, by neuronal interaction in the brain [50].

Long-chain acids, on the other hand, contribute similarly. Phytic acid, a compound abundantly identified in corn, which is used as a common raw material for both baijiu and whisky production, can be bio-transformed to cyclohexanol during microbial fermentation. This compound, however, offers many health benefits, including mitigating fatty liver, hepatitis and liver cirrhosis, and hyperlipidemia [58].

However, it should be noted that certain health potentials of the abovementioned compounds are investigated under in vitro assays, and that ethanol matrix-based experiments are required for more comprehensive results. In addition, the detoxification or mitigation of ethanol damage of these compounds is also among the top research interests.

#### 6.1.2. Phenolic Compounds

Phenolic compounds are one of the groups well known for their antioxidant capacity. For both spirits, these compounds are derived from grains as raw materials—microbial bioconversion for baijiu during fermentation, and extraction from oak barrels for whisky maturation. Diverse research approaches are trialed, including in vitro and in vivo assays and clinical investigations, to identify their potential health benefits from a broader perspective.

Phenolic compounds identified in baijiu include guaiacols as the major research target, such as 4-ethylguaiacol and 4-methylguaiacol. These compounds, as well as vanillin, presented cytoprotection against abnormal oxidative stress via in vitro analysis [91]. Research demonstrated that such phenols showed a protective capacity by gene modulation, especially the expression levels of antioxidative enzymes [91]. One of the compounds, 4-ethylguaiacol, was examined, demonstrating an anti-inflammatory effect via in vitro assay [67]. Such an effect may be explained by the inhibition on the activation of inflammasome and the production of inflammatory cytokines [67].

In whisky, human-based clinical trials were conducted, in which phenolic compounds are treated as a whole for health-related investigations. Consumption of whisky, instead of unmatured new-make spirit, significantly elevated the plasma total phenol content and antioxidant activity in healthy male participants [92]. In addition, individual groups of phenolic compounds are also investigated. Phenolic syringaldehyde, one of the key odorants in whisky, was studied and validated for its pharmaceutical values via molecular docking and subsequent in vivo and in vitro assays [93]. Health potentials, such as anti-oxidation, anti-inflammation, and anti-diabetes, are identified with syringaldehyde as the individual target, and therefore, such exploration of composite effects in the ethanol matrix is highly encouraged for validation. Further biological benefits other than anti-oxidation are identified among other phenolic compounds. Several characteristic whisky polyphenols, including ellagic acid, gallic acid, lyoniresinol, syringaldehyde, syringic acid, vanillin, hydroxymethyl furfural, sinapylaldehyde, vanillic acid, coniferylaldehyde, and protocatechuic acid, are proved as having anti-melanogenic capacity via in vitro analysis [94]. Similarly, certain phenolic fractions, especially fractions over 10, 000 molecular weight, were identified as having SOD (superoxide dismutase)-like capacity [95]. Among them, ellagic acid, gallic acid, and lyoniresinol were elucidated as the key contributors against melanogenesis [94]. Ellagic acid, on the other hand, was examined as a crucial compound for eliminating ethanol-induced damage in the stomach [96]. In vivo assays on rats demonstrated the lower irritation to gastric mucosa from whisky compared with pure ethanol, which may be due to the presence of ellagic acid, under its scavenging mechanism against oxygen and hydrogen radicals [96]. For aged whisky, where phytophenols are obtained in contact with oak barrels, four phenolic compounds, including caffeic acid, vanillin, syringaldehyde, and ellagic acid, were examined, demonstrating the ability to reduce the acetaldehyde level in the blood of mice [97]. The potential mechanism therein was explained by the inhibition of the liver alcohol dehydrogenase (ADH), thus eliminating alcohol metabolism. And, therefore, alcoholic consumption in the presence of the abovementioned phenolic compounds may have a preventive effect on alcohol-related diseases.

#### 6.1.3. Other Compounds

There are other compounds than the abovementioned groups that were identified as having potential health-related benefits, especially in baijiu. Representative components include sulfur- and nitrogen-containing compounds, such as pyrazines and peptides. Tetramethylpyrazine, one of the most abundant pyrazines identified in baijiu [6,98], has long been recognized as pharmaceutically active, which was originally identified from a traditional Chinese medicine, Ligusticum chuanxiong [99]. And, therefore, numerous targeted investigations were conducted, with its potential benefits addressed as follows: positive effects on cardiovascular and cerebrovascular diseases; in relation with anticancer and diabetic complications; protective effects on the liver and kidneys; beneficial to neurodegenerative diseases [99].

In addition, another group of nitrogen-containing complex molecules, peptides, were studied. Similar to those identified from byproducts during production, peptides were discovered in baijiu spirits, as well. Polypeptides, including peptides (Ala-Lys-Arg-Ala, Pro-His-Pro) identified from sesame-flavor baijiu and peptides identified from Qingke baijiu (Val-Val-Thr-Gly-Val-Gly-Gly-Gln, Leu-Pro-Val-Gly-Pro, Leu-Leu-Ser-Pro-Pro, Phe-Pro-Leu-Gln-Pro-His-Gln-Pro), were investigated for their health-related functions via in vitro assays. Effects of these peptides include cytoprotective capacity against oxidative stress and inhibition of ACE and, therefore, potentially helpful in cardiovascular diseases [100,101,102]. However, these studies focused primarily on in vitro tests, with pure compound applied as the research target. And, therefore, more in-depth research in the context of ethanol is required to further elucidate their potential benefits.

### 6.2. Overall Effects

With the diverse compounds identified in both spirits being elucidated for their intrinsic health values, demands for the integrated effects in the ethanolic context greatly increase for both theoretical and industrial reasons. Several studies were carried out on either animal models or human volunteers, with certain aspects investigated for health potentials or mitigations of ethanol-induced damages.

Milder alcoholic liver injuries were identified in baijiu-treated animal models (mice and rats) compared with pure ethanol treatment, and such an effect is more prominent in aged baijiu rather than fresh [103,104]. In addition, aged baijiu showed a more complex interaction with gut microbiota and the subsequent serum metabolome, shedding light on further investigation of the mechanism related to liver injury [103]. An in vivo study proved the potential link between gut microbiota regulation and alcoholic liver damage mitigation [105]. Correlations among gut microbiota, volatile production, and serum metabolites (i.e., bioenergy-related, anti-inflammatory-related) were noted with mice models, and conclusions were obtained that gut microbiota and host metabolism play key roles in regulating the relief of alcoholic liver damage [105]. For whisky, similar alleviation effects were observed for gastric mucosal injury. A protective activity for gastric mucosa, potentially due to the presence of congeners in whisky, was recorded in rat models, and yet, such protection followed a dose-dependent manner and was inhibited at higher doses (i.e., 150 mg/kg body weight) [106].

Apart from ethanol-induced damage mitigation, observations focusing on other human-related aspects were made, as well. A cross-sectional study conducted in Southern China (Guangdong province) investigated the relationship between baijiu consumption and dental health—dental caries, in particular. A protective effect of baijiu consumption against dental caries was obtained in middle-aged and elderly people via statistical analysis [107]. However, with the solid scientific explanation therein unsolved, further related research elucidating the correlations and mechanisms is encouraged.

## 7. Conclusions and Outlook

Both baijiu and whisky receive great attention and acceptance in their respective cultures. Known as the best-selling alcoholic beverage globally, baijiu is mainly consumed by the Chinese community. On the other hand, however, whisky, one of the most popular distilled spirits worldwide, enjoys its fame in diverse regions. In addition, given the established culture of spirit consumption, whisky is booming in China in recent years. Such a phenomenon, along with the unstoppable globalization, enables the potential cooperative development of these two ancient and renowned spirits. However, despite the similar raw materials as ingredients, the diverse production procedures lead to various organoleptic and compositional properties.

Based on this review, the similarities and differences in terms of flavors and potential health benefits between these two spirits are summarized comprehensively. In addition, the investigation approaches applied for both spirits are of great importance for any relevant products. Examples include the molecular sensory method used in identifying the key aroma compounds in baijiu and whisky. Tailored and targeted analysis techniques for trace compounds offering potentially significant organoleptic properties in these spirits are also in great need for further development. On the other hand, given the similar byproducts obtained for both spirits during production, future research is encouraged on more diverse utilization of similar compounds to meet the zero-carbon strategy for sustainable and responsible development. Finally, the potential health benefits, though controversial in the context of alcoholic beverages, should not be neglected for investigation, especially with such a huge consumer market. Many studies, mainly focusing on in vitro assays, were conducted as pilot investigations, shedding light on future in-depth research. Most importantly, the functional compounds identified in spirits should be evaluated in the alcoholic context at proper contents for validation. Furthermore, the synergistic effects are also encouraged for thorough investigation. Such understanding will be of guiding significance for baijiu and whisky producers to produce healthier products to meet the increasing demands for healthy food by major consumer markets.

## Figures and Tables

**Table 1:** Description and presence of sulfur-containing volatile compounds in baijiu and whisky.

Compound Name	Odor Description	Whisky	Baijiu	References
Methylsulfanylmethane/Dimethyl sulfide	Sweet corn	√	√	[2,65]
Ethylsulfanylethane/Diethyl sulfide	Garlic-like	√	√	
1-Propylsulfanylpropane/Dipropyl sulfide	Garlic, onion	√	-	
Methylsulfanylpropane/Methyl propyl sulfide	Green, leek	√	-	
Methyldisulfanylmethane/Dimethyl disulfide	Vegetable	√	√	
Propyldisulfanylpropane/Dipropyl disulfide	Green onion	√	-	
2-Methyl-1-(methyldisulfanyl) propane/Isobutyl methyl disulfide	-	√	-	
Methyltrisulfanylmethane/Dimethyl trisulfide	Onion, meaty	√	√	
2-Methylsulfanylethanol/2-(Methylthio) ethanol	Meaty	√	√	
3-Methylsulfanylpropan-1-ol/3-(Methylthio) propanol	Boiled potato	√	-	
3-Methylsulfanylpropanal/3-(Methylthio) propanol	Onion, meaty	√	-	
3-Methylsulfanylpropyl acetate/3-(Methylthio) propyl acetate	Potato	√	√	
*S*-Methyl ethanethioate/*S*-Methyl thioacetate	Cheese	√	√	
2-(Methyldisulfanyl) ethan-1-ol/3,4-Dithiapentyl alcohol		√	-	
1-Ethoxy-2-(methyldisulfanyl) ethane/3,4-Dithiapentyl ethyl ether		√	-	
2-(Methyldisulfanyl) ethyl acetate/3,4-Dithiapentyl acetate		√	-	
2-Methyl-3-(methyldisulfanyl) furan/Methyl-(2-methyl-3-furyl) disulfide	Meaty, sulfury	√	-	
Sulfane/Hydrogen sulfide	Rotten egg	√	-	
Methanethiol	Rotten cabbage	√	√	
Ethanethiol	Leek	√	√	
Ethyl 3-methylsulfanyl propanoate/Ethyl 3-(methylthio) propanoate	Pineapple	√	-	
Ethyl 2-methylsulfanyl acetate/Ethyl 2-(methylthio) acetate	Green tropical	√	-	
Thiophene	Garlic	√	√	
2-Methylthiophenone	Meaty, cooked	√	-	
2,5-Dimethylthiophene	Nutty, green	√	-	
Thiophene-2-carbaldehyde/Thiophene-2-carboxaldehyde	Benzaldehyde-like	√	-	
Thiophene-3-carbaldehyde/Thiophene-3-carboxaldehyde	-	√	-	
3-Methylthiophene-2-carbaldehyde/3-Methylthiophene-2-carboxaldehyde	-	√	√	
3-Ethylthiophene-2-carbaldehyde/3-Ethylthiophene-2-carboxaldehyde	-	√	-	
5-Methylthiophene-2-carbaldehyde/5-Methylthiophene-2-carboxaldehyde	Benzaldehyde-like	√	-	
2-Methylthiolan-3-one/Dihydro-2-methyl-3(2H)-thiophenone	Sulfur, fruity	√	-	
1-Thiophen-2-ylethanone/2-Acetyl thiophene	Nutty	√	-	
1-Thiophen-2-ylbutan-1-one/2-Butanoyl thiophene	Meaty	√	-	
1-(5-Methylthiophen-2-yl) ethenone/2-Acetyl-5-methyl thiophene	Sweet, spicy	√	-	
1-Benzothiophene	Rubbery	√	-	
1,3-Thiazole	Nutty, meaty	√	-	
2-Methyl-1,3-thiazole	Vegetable	√	-	
1-(1,3-Thazol-2-yl) ethenone/2-Acetyl-1,3-thiazole	Popcorn	√	-	
5-Ethenyl-4-methyl-1,3-thiazole/4-Methyl-5-vinyl-1,3-thiazole	Nutty	√	-	
1,3-Benzothiazole	Rubbery	√	-	
2-Methyl-1,3-benzothiazole	Rubbery, coffee	√	-	
3-Ethyl-1,3-benzothiazole-2-thione/3-Ethyl-1,3-benzothiazolethione	-	√	-	
2-(Furan-2-yl)1,3-thiazole/2-(2-Furanyl) thiazole	-	√	-	
2-Penylthiophene	Fruit, sweet	-	√	
3-Penylthiophene	Meaty, roast	-	√	
2-Methylthiophene	-	-	√	
3-Methylthiophene	Plastic, sulfurous	-	√	
3-Acetylthiophene	-	-	√	
2,4-Dimethylthiophene	-	-	√	
2-Thiophenecarboxaldehyde	Almond, cherry	-	√	
Ethyl 2-thiophenecarboxylate	-	-	√	
3-Methyl-2-thiophenecarboxaldehyde	-	-	√	
Ethyl 3-thiopheneacetate	-	-	√	
3-Thiophenecarboxaldoxime	Grass	-	√	
1-(3-Methylthiophen-2-yl) ethanone	Nut	-	√	
5-Methyl-2-thiophenecarboxaldehyde	Cherry, sweet	-	√	
2-*tert*-Butyl-thiophene	-	-	√	
3-Methyl-1-benzothiophene	-	-	√	
2,5-Dimethylbenzothiophene	-	-	√	
3-Thiophenecarboxaldehyde	-	-	√	
2-Acetylthiophen	-	-	√	
1-(2-Thienyl) propanone	-	-	√	
2-Phenylthiophene	-	-	√	
Thianaphthen	-	-	√	
Benzo[c]thiophene	-	-	√	
4-Methyldibenzothiophene	-	-	√	
2-Methylbenzo[b]thiophene	-	-	√	
2,7-Dimethylbenzo[b]thiophene	-	-	√	
Dimethyl tetrasulfide	Cabbage	-	√	
Diethyl disulfide	Onion, sulfurous	-	√	
Divinyl sulfide	-	-	√	
Allyl methyl sulfide	-	-	√	
Methyl benzyl sulfide	-	-	√	
Allyl propyl disulfide	Sulfurous	-	√	
Propyl disulfide	Onion	-	√	
Diallyl disulfide	Garlic	-	√	
Isopropyl disulfide	-	-	√	
Isopropyl phenyl sulfide	-	-	√	
Difurfuryl disulfide	Coffee, roasted	-	√	
Ethyl methyl disulfide	Onion, sulfur	-	√	
Methyl *sec*-butyl disulfide	-	-	√	
Furfuryl methyl sulfide	Garlic, vegetable	-	√	
Isopropyl methyl disulfide	-	-	√	
Methyl pentyl disulfide	-	-	√	
Dipropyl trisulfide	-	-	√	
Diallyl trisulfide	Garlic	-	√	
Methyl propyl trisulfide	-	-	√	
Allyl methyl trisulfide	-	-	√	
Thiazole	Nut, sulfur, stink	-	√	
4-Methylthiazole	-	-	√	
2-Ethoxythiazole	-	-	√	
Benzothiazole	Gasoline, leather, roasted, smoky, and rubber	-	√	
2-Mercapto-4-phenylthiazole	-	-	√	
2-Methylmercaptobenzothiazole	-	-	√	
4-Methyl-5-vinylthiazole	Cocoa, roast, soil	-	√	
2-Ethyl-4-methyl thiazole	-	-	√	
1,2-Benzisothiazole	-	-	√	
4,5-Dimethyl-2-isopropyl-thiazole	-	-	√	
4,5-Dimethyl-2-isobutylthiazole	-	-	√	
Methionol	Rubber stink, cooked vegetable, gasoline	-	√	
Methioninol	-	-	√	
3-Mercapto-3-methylbutanol	-	-	√	
3-Mercaptohexanol	Passion fruity, grapefruit	-	√	
4-(Methylthio) phenol	-	-	√	
Furfuryl mercaptan	Roasted sesame	-	√	
1-Hexanethiol	Baked taste	-	√	
1-Heptanethiol	Truffle, mushroom	-	√	
2-Methyl-3-furanthiol	Beefy, vitamin, sulfur	-	√	
2-Propene-1-thiol	Onion, garlic	-	√	
2-Hydroxy-1-ethanethiol	Unpleasant	-	√	
4-Methylbenzenemethanethiol	-	-	√	
Benzenemethanethiol	Roasted	-	√	
2-Methylundecane-2-thiol	-	-	√	
Ethyl thioacetate	Onion	-	√	
*S*-Methyl butanethioate	Cheesy, cabbage	-	√	
*S*-Methyl thiohexanoate	-	-	√	
*S*-Furfuryl thioacetate	-	-	√	
*S*-Methyl propanethioate	-	-	√	
*S*-Allyl thiopropionate	-	-	√	
Methyl 2-thiolfuroate	-	-	√	
Ethyl (methylthio) acetate	Garlic, sulfurous	-	√	
3-Mercaptohexyl acetate	Stink/passion fruity, grapefruit, citrus	-	√	
Ethyl 2-mercaptoacetate	Cooked vegetable, nutty, pineapple	-	√	
Ethyl 3-methylthiopropionate	Cabbage, grassy	-	√	
Methyl 2-(methylthio) acetate	-	-	√	
Methional	Cooked potato	-	√	
3-(Methylthio) butanal	-	-	√	
Furfuryl methyl disulfide	Roast, smoke	-	√	
2-Methyl-5-(methylsulfanyl) furan	Mustard, onion	-	√	
2-Methyl-3-(methyldisulfanyl) furan	Meat-like, roast	-	√	
1-Methyl-3-[(2-methylpropyl)thio] benzene	-	-	√	
*S*-methyl methanethiolsulfonate	-	-	√	
2-Methyl-3-(methylthio)-1-propene	-	-	√	
Ethylene trithiocarbonate	-	-	√	
4,5-Dihydro-3(2H)thiophenone	Onion	-	√	
2-Acetamido-5-methyl-1,3,4-thiadiazole	-	-	√	
3-Ethylthiophene	-	-	√	
2,5-Dimethyl-1,3,4-trithiolane	-	-	√	
1,2,4-Trithiolane	-	-	√	
bis(2-Methyl-3-furyl) disulfide	Meaty	-	√	

√: such compound has been reported in the respective spirit; -: such compound has not been reported in the respective spirit.

**Table 2:** Extraction and detection methods for baijiu and whisky analysis.

Matrix	Pre-treatment Method	Analysis Methods	Compounds Analyzed	Flavor Contributions	References
Sauce-aroma baijiu	SPE, HPLC separation	UPLC-MS, NMR	6-(2-formyl-5-methyl-1H-pyrrol-1-yl)hexanoic acid, 2-hydroxymethyl-3,6-diethyl-5-methylpyrazine	Retronasal burnt aroma	[66,67]
	SBSE	UPLC-MS, GC-MS, GC-FPD, GC-NPD, GC-O,	Sulfur and nitrogen compounds, esters, ketones, aldehydes, amino acids	Important aroma compounds	[47,48]
	HS-SPME, LLE	GC-MS, GC-O, GC-FID, GC×GC-TOFMS	Sulfur compounds, esters, aldehydes, ketones, nitrogen compounds, alcohols, furans, acids	Important aroma compounds	[68]
Baijiu, general	derivatization	GC-MS	Non-volatile organic acids	Complex matrix effect	[38]
	LLE	GC-MS, GC×GC-TOFMS, GC×GC-SCD, AEDA, aroma reconstitution and omission experiments	3-Mercaptohexanol, 4-methyl-4-mercapto-2-pentanone	Tropical fruit	[69]
	derivatization	UPLC-MS/MS, UPLC-Q-TOFMS	Volatile thiols	Important aroma compounds, fruity character	[70]
	derivatization	UHPLC-HRMS	Carboxyl compounds		[71]
Feng-aroma baijiu	Direct injection	UHPLC-Q-Orbitrap, AEDA, aroma reconstitution and omission experiments	Acids, alcohols, aldehydes, ketones	Responsible for the honey aroma during aging	[18]
Light-aroma baijiu	DI, LLE, HS-SPME	GC-MS, GC-FID, GC-O, AEDA, aroma reconstitution and omission experiments	Esters, acids, alcohol, phenols, aldehydes, acetals, ketones, sulfur compounds, pyrazines	Key aroma compounds	[35,36,39]
Strong-aroma baijiu		GC-MS, FT-IR spectrometer	Esters, alcohols, acids	Important	[72]
	HS-SPME, LLE	GC-MS/O, GC-MS	3-Methylindole	Mud-like off odor	[73]
Sesame-aroma baijiu	derivatization	LC-MS/MS, aroma reconstitution and omission experiments	Benzenemethanethiol	Important contribution to roasted aroma	[74]
	LLE, DI, VSLLME (vortex-assisted surfactant-enhanced emulsification liquid–liquid microextraction), derivatization	GC-MS, GC-FID, aroma reconstitution and omission experiments	Esters, alcohols, aromatics, phenols, furans	Aroma active compounds	[43]
	LLE, HS-SPME	GC×GC-TOFMS, GC-MS, GC-O, GC-FID, AEDA	Esters, alcohols, acids, aldehydes, acetals, ketones, sulfur and nitrogen compounds, heterocycles, alkanes, other aromatic compounds	Important aroma compounds	[8,40,41,42]
Herbaceous-aroma baijiu	HS-SPME, SPE, SBSE	GC×GC-TOFMS	Esters, alcohols, acids, aldehydes, ketones, terpenes, sulfides		[68]
Laobaigan-aroma baijiu	HS-SPME	GC×GC-SCD, AEDA	Volatile sulfur compounds	Aroma active trace compounds	[75]
Jian-aroma baijiu	LLE, HS-SPME	GC-O-MS/Osme	Esters, alcohols, acids, sulfur and nitrogen compounds, aldehydes, ketones	Aroma active compounds	[45]
Scotch malt whisky	LLE	MDGC-MS-O	*E*,*Z*-2,6-nonadienal, nonan-2-ol, 4-hepten-1-ol, *E*-2-nonenal, 1-octen-3-ol	Green note compounds	[65]
		UV-HSI, hyperspectral imaging, SWIR-HSI, short-wave infra-red	Phenolic compounds	Responsible for smoky aroma	[76]
Scotch whisky	HS-SPME	GC-_IT_MS (ion trap mass)	Esters, alcohols, acids, carbonyl compounds, monoterpenols, C_13_ norisoprenoids, volatile phenols	Volatile compounds	[77]
American whisky		UHPLC-QTOF-MS/MS	Fatty acids, fatty acid lipids, phenolic compounds	Nonvolatiles	[78]
Tennessee whiskey	SAFE, SPE	GC-O, GC-MS, AEDA	Esters, ketones, alcohols, acids, aldehydes, sulfur compounds, furans, nitrogen compounds, alkanes,	Volatiles	[28]
Bourbon whisky		TD-HRGC-SIDA, (Two-dimensional high resolution) TD-HRGC-MS, GC-FID	Alcohols, alkanes, esters, ketones, aldehydes, phenols,	Potent volatiles	[32]
Whisky, general	HS, LLE, HS-SPME, HS Tenax full evaporation dynamic, SAFE	GC-MS, GC-FPD, GC-SCD, MDGC-MS, MDGC-ECD, MDGC-SCD, MDGC-NTD	Volatile sulfur compounds		[65]
	SAFE, HS-SPME, SBSE, SPME arrow	GC-MS, GC×GC-TOFMS	Esters, alcohols, nitrogen heterocyclic compounds, terpenes, acids, alkanes, aldehydes, phenols, lactones,	Volatiles	[31,34,79,80]
		FT-ICR-MS (Fourier transform ion cyclotron resonance), UHPLC-QTOF-MS/MS	Flavonols, oligolignols, fatty acids, polyphenol glycosides	Chemical signatures for barrel aging	[22]

**Table 3:** Biomedical potentials of wastes during baijiu and whisky production.

Spirit Type	Waste Type	Functional Compounds/Groups	Identified Functions	Test Method	References
Baijiu	*jiuzao*	Tripeptide Val-Asn-Pro	Antioxidant; eliminate excessive oxidative stress and activate antioxidant enzymes	In vivo	[81]
		Peptides (amino acid sequences KLPDHPKLPK and VDVPVKVPYS)	Anti-inflammatory activity	In vitro, LPS (lipopolysaccharide)-stimulated RAW264.7 macrophage cells	[82]
		Peptides (amino acid sequences AYI, AYL, DREI, DREL)	Anti-oxidation activity	In vitro assay and AAPH-induced HepG2 cells	[83]
		Peptide fractions	Anti-oxidation activity	In vitro, justified by DPPH radical (hydroxyl, superoxide anion, and nitric oxide) scavenging activity	[82]
		Peptide fractions	ACE (angiotensin converting enzyme) inhibitory effect	In vitro assay	[84,85]
		Phenolic acid compounds (vanillic, chlorogenic, *p*-coumaric, sinapic, caffeic, ferulic, and syringic acid)	Antioxidant activity, CML (N^ε^-carboxymethyllysine) inhibition in dairy models	In vitro, inhibitory effects on CML formation and radical (glyoxal) scavenging	[86]
		Melanoidins	Anti-oxidant activity, ACE inhibitory effect	In vitro assay, 2,2′-azino-bis(3-ethylbenzothiazoline-6-sulfonic acid)(ABTS^·+^), ferric-reducing antioxidant power (FRAP), 1,1-diphenyl-2-picrylhydrazyl radical (DPPH) assays	[87]
Whisky	draff	P-coumaric, rosmarinic, chlorogenic, vanillic, protocatechuic, 4-hydroxy benzoic, caffeic acids	Antioxidant activity	In vitro assay, DPPH free radical assays	[88]
Baijiu	*huangshui*	Polysaccharides HSP-3 and HSP-W	Immunomodulatory activity	THP-1 cells: induce NO and ROS production (HSP-2, HSP-3, and HSP-W); induce production of IL-1β, IFN-γ,TNF-α, and IL-6 (HSP-2, HSP-W); enhance pinocytic and phagocytic activities (HSP-2, HSP-3, and HSP-W); upregulate mRNA and protein expressions of these cytokines (HSP-2, HSP-3, and HSP-W).	[89,90]

## Data Availability

Data are contained within the article.
